# Flow diversion for unruptured MCA bifurcation aneurysms: comparison of p64 classic, p64 MW HPC, and p48 MW HPC flow diverter stents

**DOI:** 10.3389/fneur.2024.1415861

**Published:** 2024-08-14

**Authors:** V. Hellstern, N. Brenner, A. Cimpoca, P. Albina Palmarola, E. Henkes, C. Wendl, H. Bäzner, O. Ganslandt, H. Henkes

**Affiliations:** ^1^Neuroradiologische Klinik, Neurozentrum, Klinikum Stuttgart, Stuttgart, Germany; ^2^Institut für Röntgendiagnostik, Zentrum für Neuroradiologie, Universitätsklinikum Regensburg, Regensburg, Germany; ^3^Neurologische Klinik, Neurozentrum, Klinikum Stuttgart, Stuttgart, Germany; ^4^Neurochirurgische Klinik, Neurozentrum, Klinikum Stuttgart, Stuttgart, Germany; ^5^Medizinische Fakultät der Universität Duisburg-Essen, Essen, Germany

**Keywords:** flow diversion, MCA bifurcation aneurysm, SAPT, DAPT, HPC

## Abstract

**Background:**

MCA bifurcation aneurysms pose treatment challenges because of the complex hemodynamics at the bifurcation and the risk of rupture. FDS implantation has been controversial and there are only limited reports. Therefore, the aim of this study was to assess the efficacy and safety of this treatment strategy using p64 MW HPC and p48 MW HPC FDSs for MCA bifurcation aneurysms, compared with the p64 classic FDS.

**Materials and methods:**

We retrospectively analyzed our institutional database and identified all patients with saccular, non-ruptured MCA bifurcation aneurysms treated with p64 MW HPC, p48 MW HPC, or p64 classic FDS implantation alone. Aneurysms with implantation of additional devices in the same session, previous treatments, and acutely ruptured and fusiform aneurysms were excluded.

**Results:**

A total of 79 aneurysms met the inclusion criteria: 23 receiving a p64 MW HPC, 34 receiving a p48 MW HPC, and 22 receiving a p64 classic FDS. The occlusion rate was highest for the p48 MW HPC 2 mm FDS, at 88.9% at FU2, compared with 72.2% for the p64 MW HPC and 70.6% for the p64 classic. The time to aneurysm occlusion was shortest with the p64 MW HPC, at 178.31 days. The highest retreatment rate was observed with the p48 MW HPC 3 mm.

**Conclusion:**

Treatment of MCA bifurcation aneurysms with a p48 MW HPC 2 mm or p64 MW HPC FDS is a safe and reliable strategy achieving high aneurysm occlusion rates - attributable to their lower porosity in relation to the parent vessel diameter as compared to the p48 MW HPC 3 mm FDS-, with reasonable morbidity and mortality.

## Introduction

Middle cerebral artery (MCA) bifurcation aneurysms are challenging to treat because of their complex hemodynamics and inherent rupture risk. Various treatment modalities — such as surgical clipping and endovascular coiling, stent or balloon-assisted coiling, and intrasaccular devices such as the Woven EndoBridge embolization system (MicroVention Inc., Aliso Viejo, CA, United States) or the Contour device (Stryker, Kalamazoo, MI, United States) — have demonstrated suboptimal results and increased procedural risks as compared to other locations, particularly for wide-necked and large aneurysms at the bifurcation site (1, 2). In recent years, flow diverter stents (FDSs) have emerged as a promising therapeutic option for MCA bifurcation aneurysms, with the goal of altering hemodynamics and thus leading to aneurysm thrombosis without requiring the aneurysm itself to be touched during the procedure (3, 4).

Despite the potential benefits of FDSs for the treatment of MCA bifurcation aneurysms, substantial concerns remain regarding the safety and long-term outcomes of this treatment strategy. The main concerns are ischemic complications due to side branch occlusion and delayed aneurysm occlusion (4, 5). Despite growing interest in this treatment strategy, only limited data have been published, often involving small patient cohorts.

The aim of this study was to evaluate the safety and efficacy of the p64 classic FDS, p48 MW HPC FDS, and p64 MW HPC FDS (all from WallabyPhenox, GmbH, Bochum, Germany) in terms of ischemic complications and occlusion rates, and to identify factors predicting and preventing treatment failure.

## Materials and methods

### Patient population

We retrospectively reviewed our prospectively maintained institutional database to identify all patients with saccular unruptured MCA bifurcation aneurysms treated with at least one p64 classic FDS, p48 MW HPC FDS or p64 MW HPC FDS between April 2012 and June 2023. Aneurysms treated with prior or concomitant saccular or bifurcation devices (e.g., coiling, intraaneurysmal flow diversion, and bifurcation stents) or prior parent vessel implantation were excluded. Fusiform, dissecting or blister aneurysms were excluded. Patient demographic data, anatomic characteristics, procedural and post-procedural complications, diffusion-weighted imaging (DWI) lesions on post-procedural magnetic resonance imaging (MRI), and clinical and angiographic outcomes were analyzed.

MCA bifurcation aneurysms are one of the most common locations for aneurysmal rupture (6). However, predicting which aneurysms are likely to rupture remains challenging and most ruptured aneurysms tend to be small to medium in size, which has been published before, e.g., in the Barrow-trial or the ISAT trial (7, 8) and which is also corroborated by a 10-year review conducted at our institution (6).

Given these observations, our institutional protocol mandates a thorough discussion of all potential treatment options for aneurysms—endovascular intervention, microsurgical clipping, and conservative “wait and see” management—with every patient. These discussions encompass the risks and benefits associated with each approach. Final treatment decisions are made and approved by a multidisciplinary board comprising neuroradiologists, neurosurgeons, and neurologists, ensuring a comprehensive and balanced assessment for each case.

### Endovascular procedure

All patients provided written informed consent at least 24 h before the procedure.

All procedures were performed under general anesthesia through the use of a biplane digital subtraction angiography (DSA) (Axiom Artis, Siemens, Erlangen, Germany). Either a 6F or 7F short sheath with an appropriate guide catheter was used as a standard approach via the right femoral artery. An intermediate catheter was used in cases of known dilatation of the cervical vasculature or at the discretion of the treating physician. All flushing solutions were heparinized (5,000 international units (IU)/mL unfractionated heparin). Heparin was administered intravenously after inguinal puncture (average of 3,000 IU intravenous unfractionated heparin).

In most cases, the p64 classic FDS was delivered via an Excelsior XT-27 microcatheter (Stryker Neurovascular, Fremont, CA, United States). A Prowler Select Plus (Cerenovus, Irvine, CA, United States), Rapid Transit (Cerenovus, Irvine, CA, United States), Trevo18 (Stryker Neurovascular, Fremont, CA, United States), or Headway21 (MicroVention Terumo, Aliso Viejo, CA, United States) microcatheter was used to implant either a p48 MW HPC or a p64 MW HPC FDS. Implant size was selected on the basis of intraprocedural 2D and 3D calibrated measurements.

### Medication

Patients with p64 classic FDS implantation received dual antiplatelet therapy (DAPT) with clopidogrel and acetylsalicylic acid (ASA) until 2016, when DAPT was switched to ticagrelor to achieve more reliable P2Y12 receptor inhibition. DAPT was maintained at a standard dose of 75 mg clopidogrel and 100 mg ASA daily or 2× 90 mg ticagrelor and 100 mg ASA daily for 12 months. P2Y12 receptor inhibitors were then discontinued, and lifelong monotherapy with ASA was continued.

Preoperative platelet aggregation inhibition was assessed in all patients with a Multiplate Analyzer (Roche, Basel, Switzerland).

Patients treated with a p64 MW HPC FDS received single antiplatelet therapy (SAPT) with prasugrel only. Before the procedure, all patients were tested for adequate platelet inhibition with both the Multiplate Analyzer and a VerifyNow system (Accriva, San Diego, CA, United States). PRU values below 100 were considered to indicate adequate platelet inhibition. Patients were started on prasugrel 10 mg for 3 days prior to the intervention. In cases of over-inhibition, defined as PRU below 10, the prasugrel dose was switched to 5 mg and 10 mg prasugrel alternating daily, and a repeat response test was performed after 7–10 days. If the over-inhibition persisted, the dosage was reduced to 5 mg prasugrel daily. Patients were maintained on prasugrel monotherapy for 6 months and subsequently switched to lifelong daily ASA.

Patients treated with a p48 MW HPC FDS initially received the regimen intended for DAPT. After successful use of the p64 MW HPC FDS on SAPT alone, the protocol for these patients was also changed to SAPT in June 2020.

According to our institutional protocol, patients expected to have elevated risk of ischemic complications (e.g., slightly delayed perfusion of a covered branch in the final DSA run, or patients in whom we covered a branch supplying the motor cortex or in whom the FDS was undersized) received a therapeutic dose of low molecular heparin, e.g., 2 × 3,000 IU Mono-Embolex s.c. (Viatris Healthcare GmbH), daily for 4–6 weeks after FDS treatment.

### Data collection and follow-up

Patients were scheduled for clinical and angiographic very early (FU#0, 0–90 days), early (FU#1, 91–180 days), mid-term (FU#2, 181–500 days), and long-term (FU#3, >500 days) follow-up (FU). Aneurysm occlusion was graded according to the O’Kelly-Marotta (OKM) scale (9), and OKM C and D were considered to indicate adequate occlusion. Neurological examinations were performed peri-procedurally (≤24 h), post-procedurally (>24 h to 30 days), and during FU (>30 days) by a neurologist or a certified stroke nurse, and were recorded with the modified Rankin Scale (mRS) scores (10). Clinical outcomes were categorized as transient or permanent neurological deficit or death.

## Results

### Patient demographics and aneurysm characteristics

A total of 77 patients (51 female) with 79 aneurysms met the inclusion criteria. The median age was 58.6 years (±11.2 years; range 34–88 years). The mean aneurysm height was 3.1 mm (±1.2 mm; range 1.0–6.1 mm), the mean neck width was 2.8 mm (±1.2 mm; range 1.2–6.1 mm), and the mean width/neck ratio was 1.2 (±0.29; range 0.5–2.2). In 20 of the 79 aneurysms (25.3%) DAPT was administered, heparin was administered in addition to DAPT in 22 aneurysms (27.8%). SAPT alone was used in 11 aneurysms (13.9%) and heparin was administered in addition in 26 aneurysms (32.9%).

22 patients with 23 aneurysms were treated with a p64 MW HPC FDS. The median age was 59.7 years (±11 years; range 42–88 years). The mean aneurysm height was 3.6 mm (±1.5 mm; range 1.5–6.1 mm), the mean neck width was 3.4 mm (±1.2 mm; range 2.0–5.6 mm), and the mean width/neck ratio was 1.2 (±0.27; range 0.8–2.1). One device was implanted in all cases. In 15 of the 24 aneurysms (62.5%), heparin was administered in addition to SAPT.

In the p48 MW HPC FDS group, 34 patients with 34 aneurysms were treated: 10 receiving a p48 MW HPC FDS with a 2 mm diameter and 24 receiving a p48 MW HPC FDS with a 3 mm diameter. The median age was 57.9 years (±10.7 years; range 34–78 years). The mean aneurysm height was 2.9 mm (±1.1 mm; range 1.5–5.3 mm), the mean neck width was 2.9 mm (±1.0 mm; range 1.5–5.3 mm), and the mean width/neck ratio was 1.2 (±0.31; range 0.7–2.2). One device was implanted in all cases. In 19 of the 34 aneurysms (55.8%), heparin was administered in addition to the APT drug regimen.

The p64 classic FDS group included 21 patients with 22 aneurysms. The median age was 58.4 years (±11.7 years; range 36–84 years). The mean aneurysm height was 2.8 mm (±0.9 mm; range 1.5–4.9 mm), the mean neck width was 2.68 mm (±1.26 mm; range 1.2–6.8 mm), and the mean width/neck ratio was 1.2 (±0.31; range 0.5–1.7). In two cases, two FDSs were implanted, whereas in all other cases, one device was implanted. All patients were treated with DAPT; for 14 aneurysms (63.6%), heparin was added.

A summary of patient characteristics is provided in Table 1.

**Table 1:** Patients and aneurysm characteristics.

Characteristics	p64 MW HPC	p48 MW HPC	p64 Classic	Overall study population
Number of aneuryms	23/79 (29.11%)	34/79 (43.04%)	22/79 (27.85%)	79
Age in years^‡^	59.74 (11.06)	57.88 (10.7)	58.41 (11.71)	58.6 (11.2)
Female sex	15 (65.2%)	26 (76.5%)	10 (45.5%)	51 (66.2%)
Aneurysm features^‡^				
Neck (mm)^‡^	3.40 (1.19)	2.41 (ṇ)	2.68 (1.26)	2.8 (1.2)
Width (mm)^‡^	4.16 (1.58)	2.91 (1.02)	2.93 (1.24)	3.3 (1.3)
Height (mm)^‡^	3.57 (1.51)	2.98 (1.09)	2.8 (0.97)	3.1 (1.2)
Width-neck ratio^‡^	1.24 (0.27)	1.23 (0.31)	1.16 (0.31)	1.2 (0.29)

^‡^Values are means (Standard Deviations).

### Treatment and peri- and post-procedural complications

A total of 81 FDSs were implanted. A single device was implanted in the p48 MW HPC and p64 MW HPC groups. Only two patients in the p64 classic group required two FDSs; all other patients were treated with a single device. FDS implantation was performed only after confirmation of adequate P2Y12 platelet receptor inhibition and, in the case of DAPT, ASA inhibition. The FDSs were placed in the dominant vessel or if co-dominant vessels were present, in the more accessible vessel. The FDSs did not directly cover 48 aneurysms (60.8%) and directly covered 31 aneurysms (39.2%). Indirect flow diversion was performed in the majority of the p64 MW HPC group (18 of 23 aneurysms, 78.3%), whereas 10 aneurysms were treated indirectly in the p64 classic group (45.5%), and 20 aneurysms were treated indirectly in the p48 MW HPC group (58.8%).

Intra- and periprocedural complications within 24 h were noted in 6 of the 79 total aneurysms (7.6%): none in the p64 MW HPC group, two in the p48 MW HPC group (2.5%), and four in the p64 classic group (5.1%). In both cases in the p48 MW HPC group, the FDSs showed proximal fishmouthing. One case did not have delayed perfusion. In the other case, the fishmouthing caused delayed perfusion of the branch with clot formation; therefore, a Solitaire stent was temporarily deployed to fully open the FDS, and the clot was dissolved after 2 mg eptifibatide intra-arterially. Of the four cases in the p64 classic group (5.1%), two had foreshortening of the FDS after detachment, and a second FDS was implanted. In one case, the FDS could not be detached and required removal and implantation of another device. One patient experienced basal ganglia ischemia with transient mild aphasia 7 h after FDS implantation despite adequate DAPT. No intraprocedural or periprocedural hemorrhagic complications were observed.

**Figure 1:**
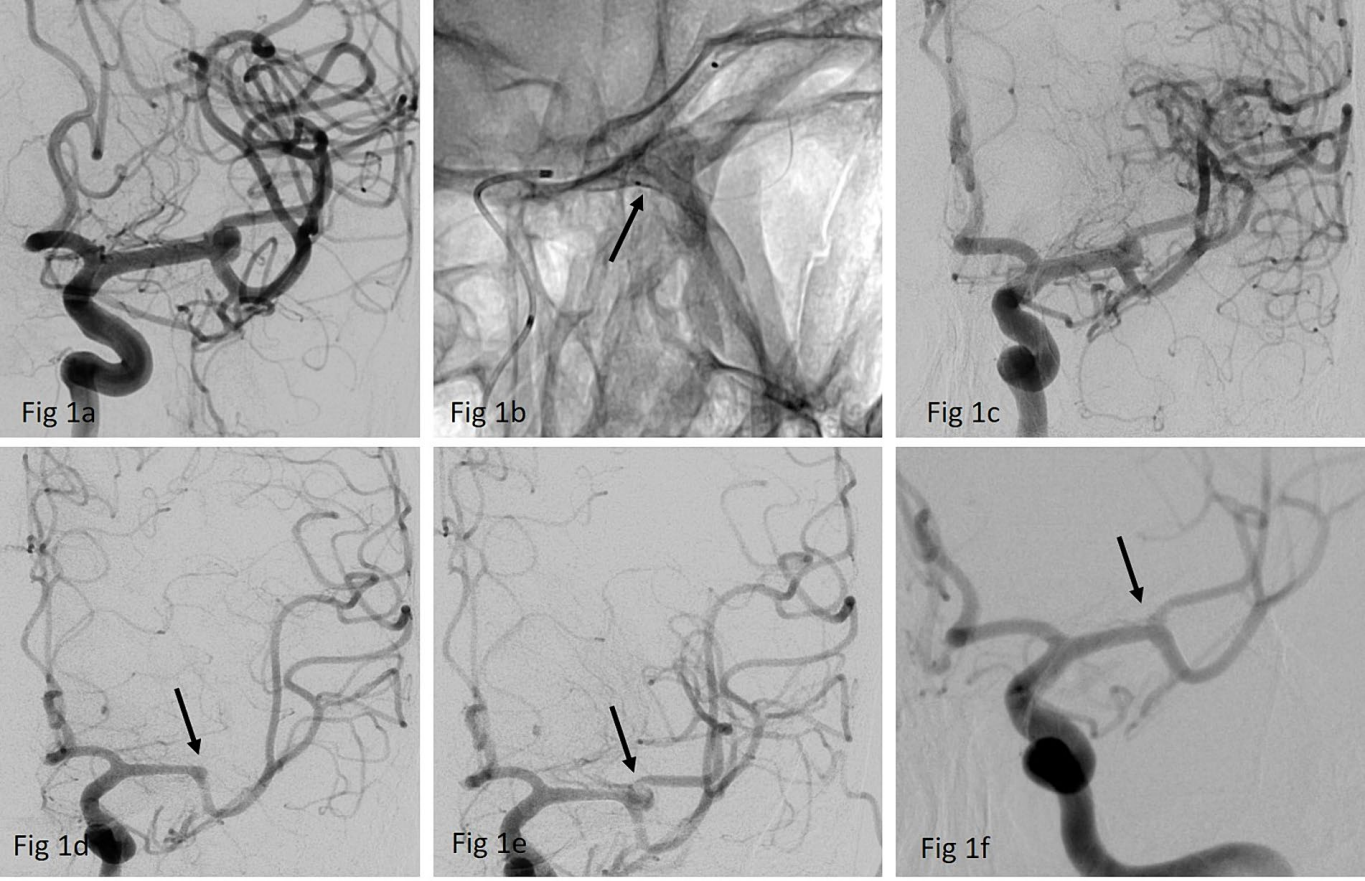
**(a)** Anterior-postero (ap) view of a left-sided MCA- bifurcation aneurysm **(b)** implantation of a p64 MW HPC 3/15 mm FDS (arrow) over a Prowler Select Plus 45° microcatheter **(c)** final run after der FDS implantation confirmed patency of all branches without any delayed perfusion; discharge medication regimen was 1 × 10 mg prasugrel p.o. daily and 2 × 3,000 I.U. of MonoEmbolex s.c. for 2 weeks **(d)** DSA after 10 days: patient presented with a right- sided hemiparesis and global aphasia- NIH 14 due to occlusion of the jailed branch (arrow). Multiplate and Verify Now results showed insufficient platelet inhibition as the patients had discontinued the prescribed regimen. **(e)** Recanalization of the jailed branch (arrow) was achieved with intra-arterial infusion of eptifibatide and aspiration within the FD. Patient made a good clinical recovery, NIH at discharge was 1 due to a mild residual aphasia **(f)** 3 months DSA FU shows a complete occlusion of the aneurysm while the jailed superior trunk remained patent (arrow).

Post-procedural complications (24 h to 30 days) were observed in 12 of the 77 patients (15.6%) in the three FDS groups: seven patients in the p64 MW HPC group (30.4%), two patients in the p48 MW HPC group (5.9%), and three patients in the p64 classic group (13.6%). Of the seven patients in the p64 MW HPC group, one patient had an asymptomatic SAH on MRI before discharge, and one patient presented with transient right-hand paresis due to aseptic encephalitis, which was treated with steroids. One patient presented with basal ganglia ischemia, and one patient presented with ischemia secondary to an occluded branch. Two patients experienced in-stent thrombosis because of noncompliance with the drug regimen, and were treated with mechanical thrombectomy or intra-arterial infusion of eptifibatide (Figure 1). One patient had a complete collapse of the FDS that could not be recanalized, thereby resulting in a large MCA infarct. In total, four permanent neurological deficits were found, including only one change in the mRS score from 0 to 5. The other three patients had only minor neurological deficits (one patient with an mRS score change from 0 to 2, and two patients with an mRS score change from 0 to 1).

Of the two patients in the p48 MW HPC group with post-procedural complications, one patient did not adhere to the prescribed SAPT and presented with aphasia due to ischemia in the basal ganglia and jailed branch, whereas the FDS remained completely patent (mRS score change from 0 to 1). One patient with a Chiari-1 malformation experienced sudden unexpected death 3 days after the procedure. An autopsy revealed no treatment- or device-related cause of death.

In the p64 classic FDS group, one patient had an asymptomatic left frontal MCA infarct in the territory of the jailed branch, and two patients had symptomatic ischemia due to basal ganglia ischemia, both with confirmed adequate DAPT and patent FDS on DSA. Both patients had mild permanent neurological deficits (mRS score change from 0 to 2).

No delayed complications (>30 days) were observed in this patient population.

In summary, 13 of the 77 patients developed peri- or post-procedural complications, including three patients with only transient neurological deficits. Eight patients (10.4%) developed permanent neurological deficits, two patients experienced severe neurological deficits or death, and six patients experienced a shift in mRS score from 0 to ≥1 or 2. Two patients remained asymptomatic.

Pre-discharge MRI data were available for all patients. Overall, 38 MRIs did not show any DWI lesions (48.1%), and 27 MRIs showed one to five DWI lesions (34.2%). In the p64 MW HPC group, 11 MRIs showed no DWI lesions (47.8%), six MRIs showed fewer than five or five DWI lesions (26.1%), and an additional five MRIs showed more than five DWI lesions (21.7%). One MRI scan indicated a post-procedural SAH. No territory infarct was observed. In the p48 MW HPC FDS-treated group, 14 MRIs had no DWI lesions (41.2%), 17 MRIs had five or fewer than five DWI lesions (50%), and three MRIs had more than five DWI lesions (8.8%). No SAH or territorial infarcts were observed in this group. In the classic p64 FDS, 13 MRIs showed no DWI lesions (59.1%). Fewer than five DWI lesions were detected in four MRIs (18.2%), whereas no MRIs showed more than five DWI lesions. However, territorial infarcts were observed in five examinations (22.7%). No SAH or ICH was observed.

A summary of the periprocedural and post-procedural complications, and the DWI lesions on discharge MRIs are shown in Table 2.

**Table 2:** Drug regimen, peri- and post-procedural complications and DWI lesions on discharge MRIs.

	p64 MW HPC	p48 MW HPC 2 mm	p48 MW HPC 3 mm	p64 classic
Drug regimen				
DAPT	0	4 (40.0)	8 (33.3)	8 (36.4)
SAPT	8 (34.8)	1 (10.0)	2 (8.3)	0
DAPT + Heparin	0	1 (10.0)	7 (29.2)	14 (63.6)
SAPT + Heparin	15 (65.2)	4 (40.0)	7 (29.2)	0
Complications				
<24 h	0	2 (20.0)	0	4 (18.2)
24 h to 30 days	7 (30.4)	0	2 (8.3)	3 (13.6)
>30 days	0	0	0	0
Neurological deficit				
Transient	2 (8.7)	0	0	1 (4.5)
Permanent	4 (17.4)	0	2 (8.3)	2 (9.1)
Any mRS Shift	4 (17.4)	0	2 (8.3)	2 (9.1)
DWI lesions				
None	11 (47.8)	4 (40)	10 (41.7)	13 (59.1)
1–5	6 (26.1)	4 (40)	13 (54.2)	4 (18.2)
>5	5 (21.7)	2 (20)	1 (4.2)	0
SAH	1 (4.3)	0	0	0
Territorial infarct	0	0	0	5 (22.7)

## Angiographic results

### Overall

Results of very early FU#0 DSA (FU 0–90 d) were available for 15 of 79 aneurysms (19.0%). The angiographic results for the 15 aneurysms were as follows: complete or near complete occlusion (OKM C + D) in 7 aneurysms (46.7%), subtotal aneurysmal filling (OKM B) in two aneurysms (13.3%), and no changes (OKM A) in the remaining six aneurysms (40.0%). One side branch occlusion was observed.

Results of early FU#1 (FU 91–180 d) were available for 36 of 79 aneurysms (45.6%). The angiographic results were as follows: complete or near complete occlusion (OKM C + D) in 15 aneurysms (41.7%), subtotal aneurysmal filling (OKM B) in five aneurysms (13.9%), and no changes (OKM A) in 7 aneurysms (19.4%). One asymptomatic side-branch occlusion was observed.

Interim angiographic data (FU#2 181-500d) were available for 59 of 79 aneurysms (74.7%). Of these 59 aneurysms, complete or near complete occlusion (OKM C + D) was observed in 39 aneurysms (66.1%), whereas 16 aneurysms (27.1%) achieved OKM B. Four aneurysms (6.8%) remained unchanged (OKM A). Three asymptomatic side-branch occlusion was observed.

Late angiographic data (FU#3 > 500 d) show complete or near complete occlusion in 26 of 38 available aneurysms (68.4%). The overall average caliber reduction of the jailed branch was 29.3%. The overall mean time to aneurysmal occlusion was 206.8 ± 123.97 days.

### p64 MW HPC

Results of very early FU#0 DSA (FU 0–90 d) were available for 9 of 23 aneurysms (39.1%). The angiographic results for the nine aneurysms were as follows: complete or near complete occlusion (OKM C + D) in three aneurysms (33.3%), subtotal aneurysmal filling (OKM B) in one aneurysm (11.1%), and no changes (OKM A) in the remaining five aneurysms (55.5%). Mild In-stent stenosis (ISS) <50% was observed in one aneurysm (11.1%). No side branch occlusions were observed.

Results of early FU#1 (FU 91–180 d) were available for 8 of 23 aneurysms (34.8%). The angiographic results were as follows: complete or near complete occlusion (OKM C + D) in four aneurysms (50%), subtotal aneurysmal filling (OKM B) in three aneurysms (37.5%), and no changes (OKM A) in one aneurysm (12.5%). One asymptomatic side-branch occlusion was observed.

Interim angiographic data (FU#2 181-500 d) were available for 18 of 23 aneurysms (78.3%). Of these 18 aneurysms, complete or near complete occlusion (OKM C + D) was observed in 13 aneurysms (72.2%), whereas five aneurysms (27.8%) achieved OKM B. No aneurysms remained unchanged (OKM A). One patient had a high grade ISS >75% resulting in asymptomatic occlusion of the jailed branch. Late angiographic data are only scarely available (the last mean FU was 474 ± 135 days).

The mean time to the last FU was 474 ± 135 days. The mean time to aneurysmal occlusion was 178.3 ± 103.62 days. No patients required retreatment. Indirect flow redirection was used in 18 of 23 aneurysms (78.3%). The overall average caliber reduction of the jailed branch was 40.8% (Table 3).

**Table 3:** Average percentage of caliber reduction of the jailed branches per time interval.

Average percentage of caliber reduction of jailed arteries per time interval^‡^	p64 MW HPC	p48 MW HPC 2 mm	p48 MW HPC 3 mm	p64 classic
0–90 d	28.54% (34.91)	60% (56.57)	n.a.	29.02% (28.17)
≤180 d	24.66% (33.75)	52.5% (17.68)	12.89% (15.28)	24.48%(16.71)
≤500 d	25.92%(27.13)	47.33% (34.71)	16.87% (13.86)	32.55% (22.85)
>500 d	40.84% (38.23)	50.57%(45.65)	16.38% (20.14)	41.25% (23.3)
Caliber reduction of 90–100% of jailed branches at last follow-up	1/10 (10%)	1/4 (25%)	0/10 (0)	1/17 (5.9%)

^‡^Values are means (Standard Deviations), n.a. = not available.

### p48 MW HPC

Results of very early FU#0 DSA (FU 0–90 d) were available for 3 of 34 aneurysms (8.8%). Of these three aneurysms, angiographic results showed complete or near complete occlusion (OKM C + D) in two aneurysms (66.6%), and both FDSs were p48 MW HPC with 2 mm diameter. In the third aneurysm (33.3%), no change (OKM A) was observed, and a 3 mm diameter p48 MW HPC was implanted. One side branch occlusion was noted.

Results of early FU#1 DSA (FU 91–180 d) were available for 11 of 34 aneurysms (32.4%). The angiographic results were as follows: complete or near complete occlusion (OKM C + D) in four aneurysms (36.4%), subtotal aneurysmal filling (OKM B) in two aneurysms (18.2%), and no changes (OKM A) in five aneurysms (45.5%). All seven non-occluded aneurysms were treated with a p48 MW HPC 3 mm; the four occluded aneurysms were treated with a p48 MW HPC 2 mm in two cases or a p48 MW HPC 3 mm in the other two cases. No side branch occlusions were observed.

Intermediate FU#2 DSA results (181–500 d) were available for 24 of 34 aneurysms (70.6%). Of these 24 aneurysms, complete or near complete occlusion (OKM C + D) was observed in 14 (58.3%) aneurysms. However, considering the diameter of the p48 MW HPC, eight of nine aneurysms (88.9%) showed OKM C + D when a 2 mm diameter p48 MW HPC FDS was used, whereas only 6 of 15 (40%) aneurysms showed OKM C + D when a 3 mm diameter device was used. Moreover, 6 of 24 (25%) aneurysms achieved OKM B, and 4 of 24 aneurysms (16.7%) remained unchanged (OKM A). Again, one jailed branch occlusion was observed. No patients had relevant ISS.

A late FU#3 DSA after more than 500 days was available for 16 of 34 aneurysms (47.1%). Of these 16 aneurysms, angiographic results showed complete or near complete occlusion (OKM C + D) in eight aneurysms (50%); 3 of 3 aneurysms (100%) were occluded where a p48 MW HPC of 2 mm diameter had been implanted, and 5 of 13 aneurysms (38.5%) showed adequate occlusion where a p48 MW HPC of 3 mm diameter had been used.

**Figure 2:**
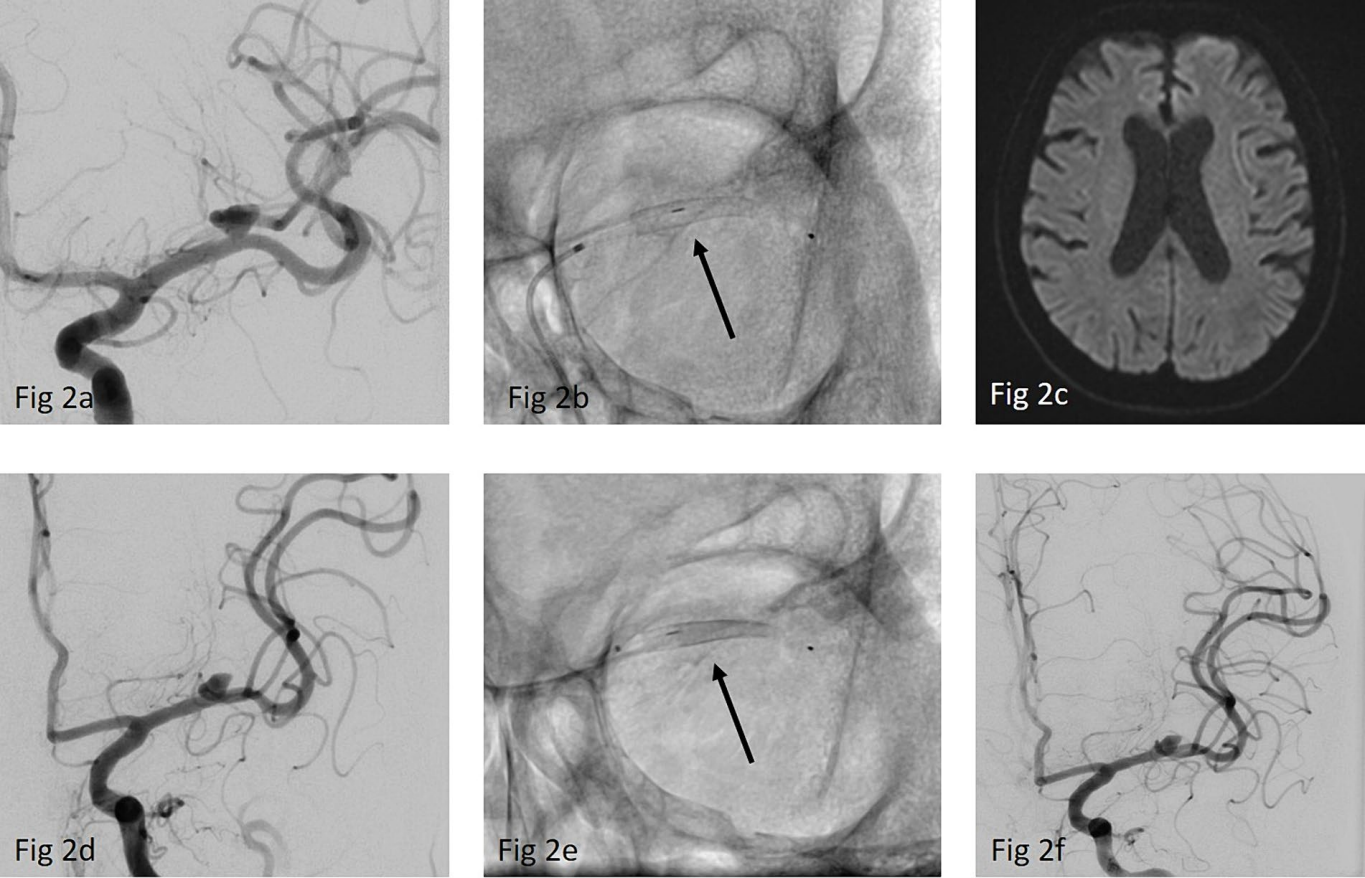
**(a)** ap-view of a left sided MCA-bifurcation aneurysm with a dominant inferior trunk **(b)** implantation of a p48 MW HPC 3/12 mm (arrow) over a Rapid Transit microcatheter **(c)** MRI prior to discharge showed no DWI lesions **(d)** DSA- FU after 12 months showed only a slight reduction of the aneurysm size while the caliber of the jailed superior trunk also remained nearly unchanged **(e)** Re-treatment of the aneurysm by implanting a second p48 MW HPC 2/15 mm (arrow) **(f)** final run after implantation of the second FDS showing no delayed perfusion of the jailed branch. FU is not yet available.

The average time to the last FU was 779.3 ± 252.48 days. The mean time to aneurysm occlusion was 189.3 ± 115.7 days. Seven patients underwent retreatment of the aneurysm by placement of a second FDS, six of whom were treated primarily with a 3 mm diameter p48 MW HPC (Figure 2). In 6 of 10 aneurysms in the 2 mm group (60%) and 14 of 24 aneurysms in the 3 mm group (58.3%), the FDS was implanted as an indirect flow diverter. The overall average caliber reduction of the jailed branch was 50.6 and 16.4%, respectively (Table 3).

### p64 classic

Results of very early FU#0 (FU 0–90 d) were available for 3 of 22 aneurysms (13.6%). Of these three aneurysms, complete or near complete occlusion (OKM C + D) was observed in two aneurysms, and one aneurysm had a score of OKM B. No side branch occlusions were observed.

The results of early FU#1 (FU 91–180 d) were available for 17 of 22 aneurysms (77.3%). Of those 17 aneurysms, the angiographic results indicated OKM C + D in seven aneurysms (41.2%), subtotal aneurysmal filling (OKM B) in nine aneurysms (53.0%), and no changes (OKM A) in one aneurysm (5.9%). No jailed branch occlusion was observed. One patient had a mild, hemodynamically irrelevant ISS.

**Figure 3:**
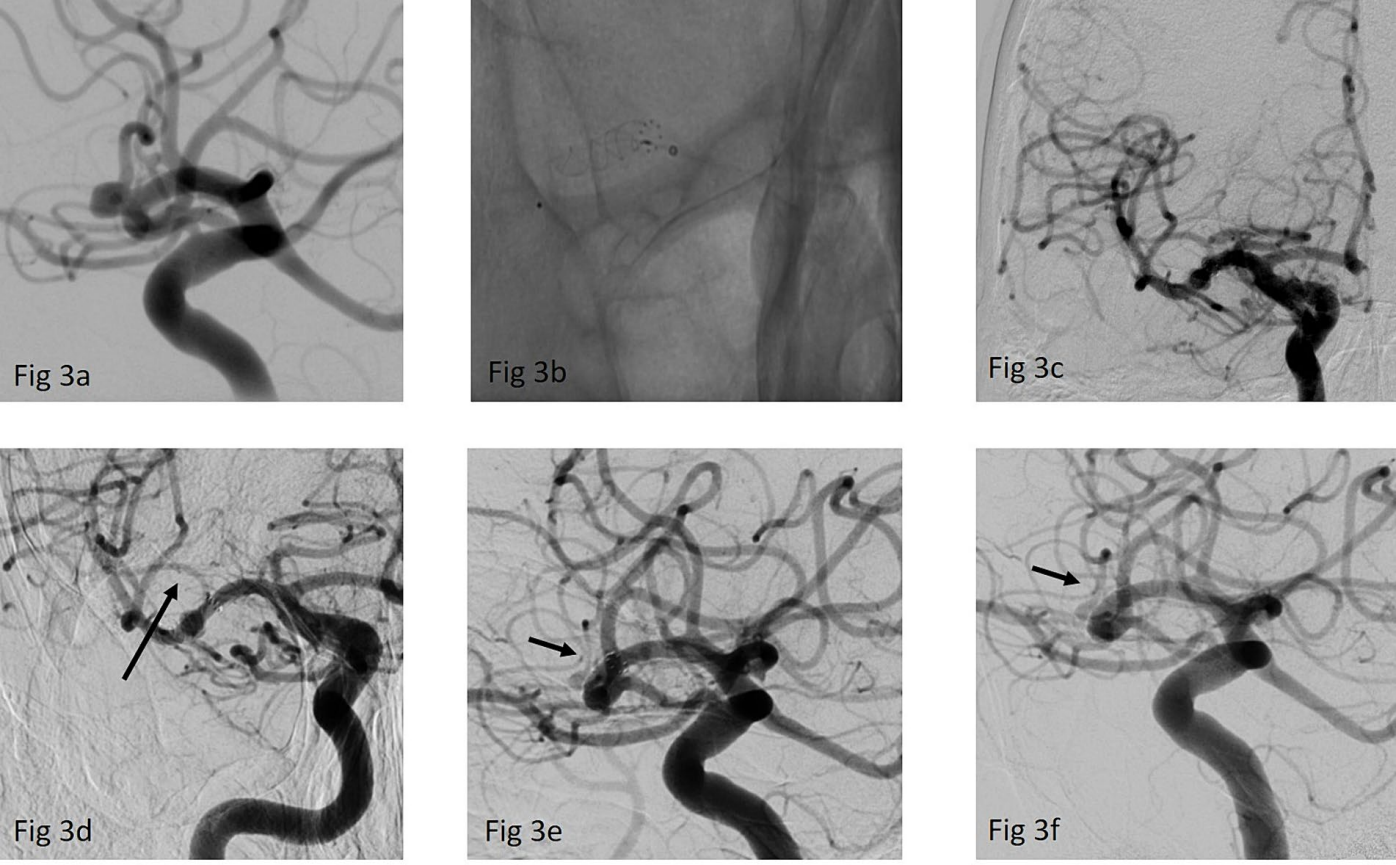
**(a)** Working projection of a right-sided MCA bifurcation aneurysm, the p64 classic FDS was intended to be placed in the dominant inferior trunk **(b)** implantation of a p64 classic 3/12 mm FDS over an Excelsior XT-27 microcatheter **(c)** ap view of the final run after the FDS implantation **(d)** first FU after 3 months showing a significant reduction of the jailed superior trunk (arrow) while the aneurysms was still perfused **(e)** second FU after 9 months confirmed a near complete occlusion of the aneurysm OKM C and further, asymptomatic caliber reduction of the superior trunk (arrow). **(f)** Last FU after 24 months showed complete exclusion of the aneurysm while the superior trunk is increasingly perfused again (arrow).

The results of intermediate angiographic data (FU#2 181-500 d) were available for 17 of 22 aneurysms (77.3%). Of these 17 aneurysms, 12 (70.6%) were classified as OKM C + D, and five (29.4%) were classified as OKM B (Figure 3). No aneurysms remained unchanged (OKM A). One side branch occlusion was observed.

Late FU#3 after more than 500 days was available for all 17 aneurysms. Angiographic results showed OKM C + D in 15 of these aneurysms (88.2%), whereas two aneurysms were graded as OKM B. Again, no new branch occlusion or relevant ISS was observed.

The mean time to the last FU was 810.12 ± 284.4 days. The mean time to aneurysm occlusion was 241.71 ± 203.2 days. The occlusion rate at the last FU was 82.4% (14/17 aneurysms). One patient required re-treatment. The rate of indirect flow diversion was 45.5% (10/22 aneurysms). The overall mean caliber reduction of the covered branch was 41.3% (Table 3).

Occlusion rates and caliber reduction of the jailed branches according to FDS are shown in Tables 3, 4.

**Table 4:** Occlusion rates per each FDS and time interval.

Aneurysm occlusion OKM C + D per time interval	p64 MW HPC	p48 MW HPC 2 mm	p48 MW HPC 3 mm	p64 classic
0–90 d	3/9 (33.3%)	2/2 (100%)	0/1 (0%)	2/3 (66.7%)
≤180 d	4/8 (50%)	2/2 (100%)	2/9 (22.2%)	7/17 (41.2%)
≤500 d	13/18 (72.2%)	8/9 (88.9%)	6/15 (40%)	12/17 (70.6%)
>500 d	3/5 (60%)	3/3 (100%)	5/13 (38.5%)	15/17 (88.2%)
Average time to aneurysm occlusion in days^‡^	178.31 (103.62)	200.11 (148.47)	173 (40.62)	241.71 (203.16)
Last follow-up in days^‡^	474 (134.99)	723.75 (173.37)	801.5 (283.08)	810.12 (284.38)
Aneurysm occlusion at last follow-up (OKM C and D)	7/10 (70%)	4/4 (100%)	4/10 (40%)	14/17 (82.4%)
Re- treatment	0	1 (10%)	6 (25%)	1 (4.5%)
FD and aneurysm				
Covered	5 (21.7)	4 (40)	10 (41.7)	12 (54.5)
Indirect covered	18 (78.3)	6 (60)	14 (58.3)	10 (45.5)

^‡^Values are means (Standard Deviations).

## Discussion

Intracranial aneurysms at the MCA bifurcation are a common yet formidable challenge in the field of endovascular treatment. Despite substantial advances in endovascular techniques and the availability of specialized devices for wide-neck bifurcation aneurysms, such as the Woven EndoBridge (MicroVention Terumo, Aliso Viejo, CA, United States), Contour (Stryker Neurovascular, Fremont, CA, United States), eCLIPS (Evasc Medical Systems, Vancouver, Canada), and pCONUS (WallabyPhenox GmbH, Bochum, Germany), these aneurysms continue to pose treatment difficulties. Complexity arises from the characteristic wide neck of the aneurysms and the incorporation of branches within the aneurysm itself (11).

Intrasaccular devices such as the WEB and Contour are potential treatment options for bifurcation aneurysms. Studies like the CERUS, WEBCAST, WEBCAST-2, and WEB-IT have reported adequate occlusion rates for these devices ranging from 79 to 84.6%, which are somewhat lower than the occlusion rates reported for FDS in our study. When considering complete occlusion rates, these figures drop to between 52.9 and 69% (12–15).

Regarding thromboembolic events, the WEBCAST study reported a rate of 17.7%, WEBCAST-2 reported 14.5%, and the CERUS study reported 11%, which are significantly higher than the rates observed in our study (12, 14, 15). Additionally, a recent study on the Contour device by Griessenauer et al. (16) reported a 6.8% rate of thromboembolic events, despite nearly 50% of cases receiving DAPT. Therefore, the perceived advantage of avoiding antiplatelet therapy with intrasaccular devices is not supported by real-world data, which show that APT is mandatory also with intrasaccular devices (16).

Moreover, the need to catheterize the aneurysm dome for the implantation of intrasaccular devices may increase the risk of procedural aneurysm injury compared to the deployment of FDSs.

The use of FDSs in the treatment of intracranial aneurysms has substantially increased since their introduction. The mechanism underlying FDS-induced aneurysm occlusion primarily involves the induction of intra-aneurysmal thrombosis via flow stasis (17). The patency of the covered branches is critical, and the extent of blood flow through the struts of a FDS is believed to depend on the flow requirements within these covered branches (18, 19). Spontaneous and often asymptomatic occlusion of a jailed branch is mainly observed in branches whose supply area has sufficient collateral supply from other territories (18). The two main arguments against flow diversion for the treatment of MCA bifurcation aneurysms address these points: the need to cover side branches with the alleged increased risk of ischemia and – in case of patency of the covered branches- the lower occlusion rates (3).

Our study may diminish these concerns, given the comparatively high occlusion rates of MCA bifurcation aneurysms treated with FDS. In particular, the p48 MW HPC 2 mm FDS showed the highest occlusion rates (88.9% adequate aneurysm occlusion at intermediate FU and 100% at late FU), followed by the p64 MW HPC (72.2% at intermediate FU) and the p64 classic FDS (70.6% at intermediate FU and 88.2% at late FU). These results are comparable to those previously reported for these FDSs (3, 20, 21). Pérez et al. (20) have reported MCA bifurcation aneurysm occlusion rates of 83 and 95% at intermediate and late FU, respectively, for the p64 classic FDS under DAPT. Hellstern et al. (21) have reported 76.5% occlusion rates for MCA bifurcation aneurysms in the first use of the p64 MW HPC in SAPT. These rates are significantly higher than those described in the literature for other FDSs (4, 22, 23). For example, Caroff et al. (22) have reported an occlusion rate of only 62%, with 43% ischemic complications observed on MRI and 21% procedural morbidity. Gawlitza et al. (4) have reported an even lower occlusion rate of 33.3% (4/12) in MCA bifurcation aneurysms at the last FU, although only short to mid-term FU data were available.

The slightly lower occlusion rate of the p64 MW HPC FDS than the p48 MW HPC 2 mm FDS might have been due to a higher proportion of patients treated with indirect flow diversion in the p64 MW HPC group. Nevertheless, the high occlusion rates of the p64 MW HPC FDS, despite the higher proportion of indirect flow diversion cases, suggest its efficacy in aneurysm closure, particularly in avoiding risky catheterization of potentially difficult-to-reach side branches. These observations are consistent with the data reported by Schob et al. (24), who observed a complete occlusion rate of 50% in short-term FU for 17 aneurysms with a mean FD-to-aneurysm distance of 1.6 mm, thus suggesting that catheterization of the more accessible branch of the MCA bifurcation appears to be equally effective as direct flow diversion. This approach not only speeds up the procedure and increases its safety, but also eliminates the need to perform risky maneuvers, such as looping to gain access to the given MCA branch.

The p48 MW HPC 3 mm FDS significantly decreased the occlusion rate, to only 38.5% at long-term FU. Correspondingly, a higher incidence of retreatment was observed. This phenomenon might be attributable to increased porosity, particularly given that the proximal M2 branches and the M1 segment are typically less than 3 mm in diameter. Consequently, stretching of the FDS increases its porosity and thus decreases its flow modulating efficacy while sparing the jailed branches or perforators (25, 26).

Regarding the patency of the occluded branches, the data in Table 3 show that the highest caliber reduction of the covered branches occurred with the lower porosity of the FDS, as observed with the p48 MW HPC 2 mm, followed by the p64 MW HPC, whereas the lowest caliber reduction was observed with the p48 MW HPC 3 mm. As expected, no side branch occlusions were observed in this cohort of patients. These differences in porosity influences the flow conditions of the jailed arteries. Notably, all cases of occlusion in the jailed branches were incidental findings on angiographic FU and thus remained clinically and MR-graphically asymptomatic.

These findings are consistent with those from previous studies by Saleme et al. (19) and Fahed et al. (27) discussing the effects of covered branches and persistent flow to jailed branches on the efficacy of flow diversion in bifurcation aneurysms. Saleme et al. (19) have suggested that symptomatic remodeling of covered side branches may depend on the extent and type of collateral blood supply. In addition, Fahed et al. (27) have suggested that persistent flow to the occluded branch may contribute to treatment failure after flow diversion. They compared flow diversion with or without occlusion of the jailed branch and found that occlusion of the jailed branch resulted in greater aneurysmal occlusion rates. Patent aneurysms were associated with leaks or holes in the neointima covering the aneurysm neck potential caused by residual flow to the jailed branch (27). In this context, the HP coating may play a role in promoting endothelialization, thus leading to faster aneurysm occlusion (28), as also our data from this patient cohort might suggest as the time to aneurysmal occlusion was significantly shorter in the HPC FDS groups as compared to the p64 classic variant.

A concern regarding MCA bifurcation FD might be the use of SAPT and the need to cover branches resulting thus potential leading to higher rates of ischemic complications. However, intra- and periprocedural ischemic complications within 24 h occurred in only 2 of 77 patients (2.6%), none of whom were in the p64 MW HPC group. Post-procedural ischemic complications were observed in 9 of 77 patients (11.7%), including three patients who did not adhere to the prescribed medication regimen. Thus, a total of six ischemic complications (7.8%) were observed in this study, despite sufficient TAH: three each in the p64 classic and p64 MW HPC groups. Similar complication rates have been reported in other studies of MCA bifurcation FDSs (20, 29, 30), and substantially higher complication rates above 15% have also been reported (31). With sufficient TAH, no ischemic complications occurred with the low profile FDS p48 MW HPC. This finding was expected, given that the higher density of the p64 FDS family and other low porosity FDSs might be more prone to ischemic complications than low profile FDSs such as the p48 MW HPC, Silk Vista Baby (Balt), or FRED jr (MicroVention). Furthermore, the specific risk of ischemic events highlights a crucial aspect that must be carefully considered in selecting the device dimensions. In our experience, oversizing the p64 classic and p64 MW HPC FDS and adding heparin for 4–6 weeks decreases ischemia risk. The relatively high proportion of patients who received low molecular weight heparin may also be an explanation why we observed a low incidence of side branch occlusions, particularly in the immediate post-interventional course, in our patient cohort, despite the implantation of FDS with low porosity. The additional administration of heparin in the first weeks after flow diverter implantation appears to favorably affect the altered flow conditions and allow a gradual adaptation to the new blood flow patterns in the branches now covered by the flow diverter stent.

Considering this seemingly beneficial influence of administering low molecular weight heparin immediately after flow diverter implantation and the fact that no hemorrhagic complications occurred in our patient cohort under this medication regimen, it is worth considering prescribing a triple anticoagulation regimen to all patients treated with flow diverter implantation for MCA bifurcation aneurysms. However, larger and prospective data are, of course, necessary in this regard.

Our comparison of DWI lesions on post-procedural MRI scans revealed a notable difference among flow diverter models. Specifically, the p64 classic had the highest proportion of MRI scans without DWI lesions with nearly 60% of the MRIs. In addition, 77.3% of MRI scans with the p64 classic showed fewer than five DWI lesions. The HPC-coated p64 and p48 FDS, which were implanted predominantly under SAPT, showed lower percentages of 47.8 and 40.9%, respectively, of MRIs scans with no DWI lesions. However, those percentages increased to 73.2 and 88%, respectively, when MRIs with fewer than five DWI lesions were considered. For example, Hellstern et al. (21) have reported fewer than five DWI lesions in 81% of discharge MRI scans when the p64 MW HPC FDS was implanted under SAPT with prasugrel for any aneurysm location in the anterior circulation. In contrast, Aguilar Perez et al. (32) have reported significantly higher DWI lesion rates exceeding 90% after implantation of a pCONUS2 HPC device under ASA SAPT, although the pCONUS (WallabyPhenox GmbH, Bochum, Germany) has significantly less material than an FDS. Of course, the use of ASA instead of a P2Y12 inhibitor is an important difference, given that P2Y12 inhibitors have significantly greater potential to inhibit platelet function (33). Notably, we found that DWI lesions were not confined to the territory of the jailed branch but also extended to other areas such as the catheterized MCA branch, thus suggesting that factors beyond device thrombogenicity, such as the deployment technique or the catheters itself might contribute to their occurrence. Overall, with respect to DWI lesions as a surrogate marker of device safety, our findings suggested that the implantation of an FDS for MCA bifurcation aneurysms is as safe as for aneurysms at other locations in the anterior circulation.

### Limitations

In summary, the primary limitations of this investigation are those inherent to retrospective data collection. Our study focused solely on our center’s experience with the implementation of three specific FDS device types. The generalizability of these findings to other FDSs remains uncertain. Moreover, whether the conclusions drawn from this study might be applicable to fusiform or ruptured aneurysms remains unclear. The use of HPC-coated flow diverter devices in the treatment of MCA bifurcation aneurysms demonstrates their potential to achieve high occlusion rates. The p48 MW HPC 2 mm had the best performance, because of its unique balance of porosity, reduced ischemic complications, and side branch protection. Whereas the p48 HPC 2 mm had commendable performance, the p48 HPC 3 mm variant presented challenges in terms of occlusion efficacy and thus requires closer examination of its retreatment rates. These findings shed light on the complex relationships among flow diverter design, dimensions, and treatment outcomes, and highlight the need for careful consideration when tailoring treatment strategies for MCA bifurcation aneurysms.

## Conclusion

In conclusion, our study demonstrated high occlusion rates and low re-treatment rates with p48 MW HPC 2 mm and p64 FDSs in treating MCA bifurcation aneurysms, thus providing a rapid and easy treatment alternative for this challenging clinical entity. These findings warrant further investigation and consideration in clinical practice.

## Data availability statement

The original contributions presented in the study are included in the article/supplementary material, further inquiries can be directed to the corresponding author.

## Ethics statement

Local ethics committee approval was obtained for the retrospective data analysis and publication (reference No. F-2018-110). The studies were conducted in accordance with the local legislation and institutional requirements. The participants provided their written informed consent to participate in this study. All patients or their legal representatives agreed to the collection, analysis, and anonymous publication of data.

## Author contributions

VH: Conceptualization, Data curation, Investigation, Writing – original draft. NB: Writing – review & editing. AC: Writing – review & editing. PA: Data curation, Formal analysis, Writing – review & editing. EH: Writing – review & editing. CW: Writing – review & editing. HB: Writing – review & editing. OG: Writing – review & editing. HH: Conceptualization, Supervision, Validation, Writing – review & editing.
